# Plerixafor Engages β-Arrestin-Dependent CXCR4 Signaling to Promote Melanogenesis via β-Catenin-MITF Activation

**DOI:** 10.3390/cimb48070730

**Published:** 2026-07-17

**Authors:** Tsong-Min Chang, Ting-Ya Yang, Huey-Chun Huang

**Affiliations:** 1 Department of Applied Cosmetology, HungKuang University, Taichung City 433304, Taiwan; ctm@hk.edu.tw; 2 Department of Medical Laboratory Science and Biotechnology, College of Medicine, China Medical University, Taichung City 40402, Taiwan; s9724053@mail.cmu.edu.tw

**Keywords:** CXCR4, β-arrestin, β-catenin, MITF, Plerixafor, re-pigmentation

## Abstract

Plerixafor is a clinically approved CXCR4 antagonist that mobilizes hematopoietic stem cells by disrupting CXCL12/CXCR4 retention signaling. However, its biochemical effects on melanocytes and pigmentation remain unexplored. We investigated how Plerixafor modulates CXCR4 signaling in melanocytes and evaluated its potential as a pro-melanogenic agent using in vitro and in vivo approaches. Human PIG1 melanocytes were treated with 10 nM Plerixafor with or without hydroquinone (HQ), followed by qPCR for MITF and tyrosinase expression, flow cytometry for CXCR4/CXCR7 and integrin profiling, transwell migration assays, β-arrestin siRNA knockdown, Western blotting, subcellular fractionation, and ChIP-qPCR for β-catenin binding to MITF regulatory regions. A murine HQ-induced depigmentation model was used to test topical Plerixafor on pigmentation, hair follicles, melanogenic gene expression, and systemic safety markers. Plerixafor significantly increased MITF and tyrosinase mRNA and enhanced melanocyte migration while counteracting HQ-induced suppression of melanogenic genes. In addition, it reduced cell-surface CXCR4 (consistent with β-arrestin-mediated receptor internalization) without altering CXCR7, c-KIT, or N-cadherin. β-Arrestin knockdown abolished Plerixafor-induced ERK phosphorylation and melanogenic responses, confirming β-arrestin dependence. Plerixafor promoted β-catenin nuclear translocation and direct β-catenin occupancy at MITF promoter/enhancer TCF/LEF motifs. In vivo, topical Plerixafor restored HQ-induced depigmentation, increased hair follicle number and melanin content, and upregulated cutaneous MITF and tyrosinase without hepatic, renal, or inflammatory toxicity. Plerixafor functions as a biased CXCR4 ligand in melanocytes, influencing the β-arrestin–β-catenin–MITF signaling axis to drive melanogenesis and re-pigmentation. These findings identify β-arrestin-dependent CXCR4 signaling as a tractable pharmacologic mechanism for therapeutic re-pigmentation in pigmentary disorders.

## 1. Introduction

Fully differentiated human melanocytes, which primarily reside in the basal layer of the skin’s epidermis, are essential for synthesizing melanin within specialized organelles and transferring these pigment-containing organelles to neighboring keratinocytes. Melanocytes respond to various signals, including UV radiation, hormones, and paracrine signaling from surrounding tissues, to regulate the amount and type of melanin produced [1]. Dysregulation of these factors can lead to pigmentation disorders in which melanocytes are lost or become dysfunctional. Research indicates that melanocytes are primarily located in reservoirs, particularly within hair follicles. However, the number of functional melanocytes and the ability to produce melanin tend to decrease with age, contributing to the lightening of skin and hair color observed in older individuals [2]. Current therapeutic strategies for pigmentation disorders include, but are not limited to, skin grafting and autologous melanocyte transplantation. However, these approaches are limited by the low proliferative capacity of melanocytes obtained from skin biopsies. Recent advances in generating melanocytes from human pluripotent stem cells have demonstrated potential, providing a more sustainable source for cell therapy [3,4]. Generating healthy melanocytes would likely involve enhancing or mimicking the signals that attract and support melanoblast/melanocyte survival and differentiation, which could potentially involve controlled manipulation of chemokine signaling [5].

Among the signaling networks involved in cell migration and tissue homeostasis, the CXCL12/CXCR4 axis has attracted noticeable attention. Plerixafor (Mozobil^®^, AMD3100, Genzyme, Cambridge, MA, USA) is a medication primarily used in medicine to mobilize hematopoietic stem cells (HSCs) [6]. It blocks the binding of stromal cell-derived factor-1α (SDF-1α, also known as CXCL12) to CXCR4 [7,8]. This disruption leads to the mobilization or release of these cells from their niche into the peripheral circulation, resulting in their release and subsequent vulnerability to external factors. The CXCL12/CXCR4 signaling axis acts as a primary homing and retention signal. CXCL12 signals primarily through two G-protein-coupled transmembrane receptors—CXCR4 and CXCR7—, but they differ in their signaling mechanisms and physiological roles [9]. Pleiotropic effects of the CXCR4/CXCR7/CXCR12 pathway have been reported in various physiopathological processes, as well as in malignant diseases [10,11]. Plerixafor is considered a biased antagonist of the CXCR4 receptor. It effectively blocks the binding of CXCL12 and prevents the activation of the G-protein signaling pathway, which is the canonical pathway responsible for the retention and survival signals within the stem cell niche [12]. Plerixafor also acts as a β-arrestin-biased agonist. Binding to CXCR4 promotes the recruitment of the β-arrestin protein that typically triggers CXCR4 internalization. CXCR7 acts as a decoy receptor, influencing CXCR4 binding to CXCL12 and thereby limiting excessive signaling, which helps maintain the balance between cell movement, survival, and differentiation [13,14].

In a previous screening of approximately 1500 FDA-approved compounds, our laboratory unexpectedly identified Plerixafor as a potent stimulator of melanin production in B16F10 melanoma cells. Recently, studies have revealed its intriguing effects on skin biology and hair follicle dynamics. Treatment with Plerixafor protects against the development of UV-induced skin cancer in murine models, highlighting the therapeutic potential of modifying this chemokine axis. Plerixafor promotes wound healing in diabetic patients and inhibits tumor proliferation [15,16,17]. In the allergic contact dermatitis mouse model [18], CXCL12 enhances itch and pain sensations while Plerixafor relieves them. Zheng et al. identified that silencing the CXCL12/CXCR4 axis stimulates hair growth in androgenetic alopecia [19]. CXCR4 expression in primary melanocytes was variable but consistent [20]. Therefore, we hypothesized that Plerixafor acts as a β-arrestin-biased CXCR4 ligand in melanocytes, affecting a β-catenin–MITF transcriptional pathway to promote melanogenesis and re-pigmentation. To test this, we combined in vitro pathway dissection in human PIG1 melanocytes with an in vivo hydroquinone-induced depigmentation model to define the CXCR4-dependent mechanisms and therapeutic potential of Plerixafor in pigmentation disorders. Hydroquinone (HQ) is a phenolic depigmenting agent that functions primarily as an alternate substrate and competitive inhibitor of tyrosinase, diverting tyrosine oxidation toward non-melanogenic quinones and thereby suppressing melanin synthesis in active melanocytes [21,22].

## 2. Materials and Methods

### 2.1. Melanocyte Culture and Treatment

Human PIG1 cells (catalogue number CRL-2208, purchased from ATCC, Manassas, VA, USA), an immortalized human epidermal melanocyte cell line, were used in this study. PIG1 cells retain key melanocytic characteristics, including melanogenesis-related signaling responses and the expression of melanocyte-associated markers, and provide a stable and reproducible model for investigating the molecular mechanisms regulating melanocyte function. As a result, PIG1 cells were selected primarily for mechanistic analyses of CXCR4-mediated signaling and downstream melanogenic pathways under controlled experimental conditions. Cells were cultured in Dulbecco’s Modified Eagle’s Medium (DMEM) supplemented with 10% fetal bovine serum (Gibco; Grand Island, Thermo Fisher Scientific, NY, USA), 100 U/mL penicillin, and 100 μg/mL streptomycin. The cells were kept in a humidified incubator (Thermo Fisher Scientific, Marietta, OH, USA) at 37 °C with 5% CO_2_, and passage cells were regularly maintained using TrypLE Express (Thermo Scientific, Waltham, MA, USA) to achieve approximately 80% confluency. PIG1 cells (1 × 10^6^ /mL) were allowed to grow for 16 h before treatment, after which they were treated with hydroquinone (1 μM) (AlfaAesar, Thermo Fisher Scientific, Waltham, MA, USA) for 8 h, followed by a replacement with Plerixafor (10 nM) (MedChemExpress, New Jersey, NJ, USA) for 16 h. The 1–10 nM concentration range, used in vitro—which approximates the plasma levels achieved clinically during stem cell mobilization and has been established as non-cytotoxic in primary cells—was selected for treating cells [23,24].

### 2.2. Cell Migration Assay

PIG1 cells were seeded at a density of 1 × 10^4^ cells per well in the upper chamber of a transwell plate with a pore size of 5 μm. The lower chamber contains a medium supplemented with Plerixafor for 24 h, allowing for migration. The number of migrated cells was fixed with methanol, stained with a 0.2% crystal violet solution, and counted under a microscope at a magnification of ×200 in five random fields per well. The migration ratio was calculated as the % of trans-migrated cells compared with the DMSO vehicle control. This assay was repeated in triplicate for each experimental condition.

### 2.3. siRNA Transfection

β-Arrestin1/2 siRNA oligonucleotides were purchased from Tri-I Biotech., Taichung, Taiwan. Their nucleotide sequences were as follows: 5′-AAAGCCUUCUGCGCGGAGAAU-3′ (β1-arrestin) and 5′-AAGGACCGCAAAGUGUUUGUG-3′ (β2-arrestin). PIG1 cells were seeded in six-well plates, and transfection was performed when the cell confluence reached approximately 60%. siRNAs (20 nM) were mixed with Lipofectamine RNAiMAX Reagent (Invitrogen, Thermo Fisher Scientific, Waltham, MA, USA) according to the manufacturer’s protocol and used for transfection. Transfection efficiency was assessed using Western blot analysis.

### 2.4. Flow Cytometry

PIG1 cells (1 × 10^6^/mL) were harvested and incubated with 100 ng of fluorescently conjugated monoclonal N-cadherin antibodies (Genetex, Irvine, CA, USA), c-kit (YR145; abcam), CXCR4 (HL2612; Genetex, Irvine, CA, USA), CXCR7 (C1C2; Genetex, Irvine, CA, USA), integrin alpha 2 (EPR5788; abcam), integrin alpha v (272-17E6; abcam), and integrin beta 1 (12G10; abcam) for 2 h. Cells were washed to remove unbound antibodies and then resuspended in PBS for analysis. A total of 30,000 events were recorded by the Attune NxT flow cytometer (Thermo Fisher Scientific, Waltham, MA, USA). For each surface marker, a positive threshold at the mean fluorescence intensity (MFI) was established based on the control sample. This threshold was then applied uniformly to all treatment conditions within the same experiment. The % of positive cells was defined as the proportion of events exceeding this threshold. Data are also presented as a fold change in MFI relative to vehicle controls. A minimum of three independent experiments was performed for statistical analysis.

### 2.5. RNA Isolation and Quantitative PCR (QPCR)

Total RNA was extracted using the TRIzol method, according to the manufacturer’s instructions. The RNA was then reverse-transcribed into cDNA using the PrimeScript RT kit (Takara Bio, Kyoto, Japan). The resulting cDNA was subjected to quantitative fluorescence PCR (qPCR) according to the guidelines of the SYBR Premix Ex Taq II kit (TaKaRa, Tokyo, Japan). RT-qPCR was conducted on a 7500 instrument (Applied Biosystems, Foster City, CA, USA). The expression level was normalized against that of 18S rRNA, and the relative transcription levels were calculated using the relative quantification method (2^−ΔΔCT^). The primers are listed in Table S1.

### 2.6. Chromatin Immunoprecipitation—Quantitative PCR (ChIP-qPCR) Assay

The ChIP-qPCR assay was performed using the SimpleChIP^®^ Plus Enzymatic Chromatin IP Kit (Cell Signaling Technology Inc., Danvers, MA, USA), according to the manufacturer’s instructions. The cells were harvested and fixed in 1% formaldehyde with vacuum infiltration, and crosslinking was reduced by adding glycine (0.125 M). The chromatin was collected, sonicated, and then immunoprecipitated using an anti-β-catenin antibody. The immunoprecipitated DNA fragments were analyzed by quantitative PCR using specific primers (Table S2). Enriched values were normalized with the level of the IgG group.

### 2.7. Western Blot Analysis

Proteins of PIG1 were immunoblotted with specific primary antibodies against ERK (1:3000; EPR17526; abcam), pERK (1:1000; EPR19401; abcam), β-catenin (1:1000; D10A8; Cell Signaling Technology, Danvers, MA, USA), β-arrestin1/2 (1:1000; D24H9; Cell Signaling Technology), β-actin (1:10,000; Genetex, CA, USA), GAPDH (1:10,000; Genetex, CA, USA), and Lamin B1 (1:5000; Genetex, CA, USA), and then incubated with the corresponding secondary antibodies. Chemiluminescence images were acquired using the Invitrogen iBright FL1000 Imaging System (Thermo Fisher Scientific, Waltham, MA, USA) in Chemi mode. Built-in push-button Smart Exposure technology was employed to optimize the signal-to-noise ratio, and the luminescence intensity of the bands was quantified by using ImageJ.

### 2.8. Animal Treatments

All animal experiments were approved by the Institutional Animal Care and Use Committee of China Medical University (Approval No. CMUIACUC-2024-115) and conducted in accordance with the International Standards on Animal Welfare. Female C57BL/6 mice were purchased from the National Center for Biomodels (NCB, Taipei City, Taiwan) and subsequently sensitized to 2% HQ by applying a DMSO solution of HQ onto shaved flanks (100 μL/mouse) for three weeks. The mice were divided into 4 groups (5 mice each) using stratified randomization. For the experimental groups, 10 mM or 0.1 mM of Plerixafor was applied daily to the backs of all mice for 14 days. Topical concentrations of 0.1–10 mM were selected based on preliminary dose-finding experiments and to achieve local tissue exposure sufficient for CXCR4 engagement without systemic effects. The control group received DMSO at the same interval. All mice were sacrificed and sampled on day 37.

### 2.9. Specimens and Immunohistochemistry

After administering pentobarbital (200 mg/kg, IP), the skin tissue (1 cm^2^) from mice was harvested, fixed in a 4% buffered neutral formalin solution for 24 h at room temperature, and then embedded in paraffin. Serial sections were floated in warm water containing 2% gelatin to prevent them from peeling off the sample. The 5 mm paraffin cross-sections were deparaffinized, rehydrated, and stained with hematoxylin and eosin (H&E) for histochemical analysis or with the Fontana–Masson Stain Kit for staining melanin. For each mouse, three non-overlapping fields per section were acquired from the interscapular area at 200× magnification, with image quantification performed using ImageJ software version 1.53c [25]. Values from individual fields were averaged to obtain a single value per animal, and one-way ANOVAs were used to compare group means with post hoc testing.

### 2.10. Inflammatory Cytokine Activity

To assess the effect of Plerixafor on proinflammatory markers, the Mouse Inflammation Antibody Array Membrane (40 targets) (Abcam 133999, Cambridge, UK) was used, following the manufacturer’s protocol. Briefly, 250 µg of serum from each sample was diluted in 1 mL of blocking buffer and applied to each array membrane. After washing, biotin-conjugated antibodies were added, followed by incubation with streptavidin conjugated to horseradish peroxidase. The membranes were then visualized with a Chemi-Doc imaging system (Bio-Rad, Hercules, CA, USA). Densitometric analysis of the signals was performed using the Protein Array Analyzer tool in ImageJ software (Research Services Branch, National Institute of Mental Health, Bethesda, MD, USA). Array data were normalized according to the manufacturer’s instructions, and heatmaps of the results were generated using Microsoft Excel 2019.

### 2.11. Statistical Analysis

All quantitative data were obtained from at least three independent experiments and are presented as the mean ± standard deviation (SD). Results were statistically evaluated using a one-way analysis of variance (ANOVA) followed by Tukey’s post hoc test or Student’s *t*-test, using the Statistical Package for the Social Sciences (SPSS) version 21 (IBM, Armonk, NY, USA). Significance thresholds were set at *p* < 0.05 *, *p* < 0.01 **, and *p* < 0.005 *** for comparisons with the control. Comparisons within the HQ group are denoted as *p* < 0.05 #, *p* < 0.01 ##, and *p* < 0.005 ### at equivalent thresholds.

## 3. Results

### 3.1. Plerixafor Enhanced Melanogenesis

**Figure 1:**
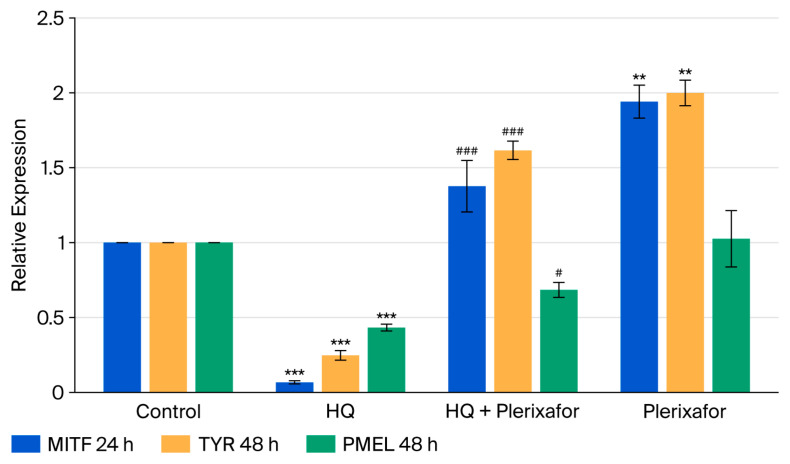
Plerixafor reverses HQ-induced suppression of melanogenic genes in PIG1 cells. PIG1 cells were treated with HQ, Plerixafor, or their combination, and MITF (24 h), tyrosinase (TYR, 48 h), and PMEL (48 h) mRNA levels were quantified by qPCR using 18S rRNA as an internal control. Relative expression levels were normalized to the control (set as 1.0) and are shown as the mean ± SD from *n* ≥ 5 independent experiments. Statistical significance was assessed by a one-way ANOVA followed by Tukey’s post hoc test. ** *p* < 0.01, and *** *p* < 0.005 versus control; # *p* < 0.05, and ### *p* < 0.005 versus HQ.

We examined the pro-melanogenic activity of Plerixafor by measuring the expression of the key melanogenic regulator MITF at 24 h, tyrosinase at 48 h, and the melanosome structural protein PMEL at 48 h using qPCR in PIG1 cells (Figure 1). Plerixafor markedly upregulated MITF and tyrosinase mRNA levels compared with the control group, while maintaining PMEL expression close to basal levels. In contrast, HQ treatment substantially suppressed MITF, tyrosinase, and PMEL transcripts, consistent with its inhibitory effect on tyrosinase activity and melanogenic gene expression; however, co-treatment with Plerixafor largely reversed the HQ-induced downregulation of MITF and tyrosinase, with only a modest restoration of PMEL expression. These findings suggest that Plerixafor stimulates melanogenesis predominantly by enhancing the MITF/tyrosinase transcriptional program, thereby promoting melanin production, and can counteract HQ-mediated transcriptional suppression even though it may not directly relieve enzymatic inhibition of tyrosinase.

### 3.2. Effects of Plerixafor and HQ on PIG1 Cell Migration and Surface Marker Expression

**Figure 2:**
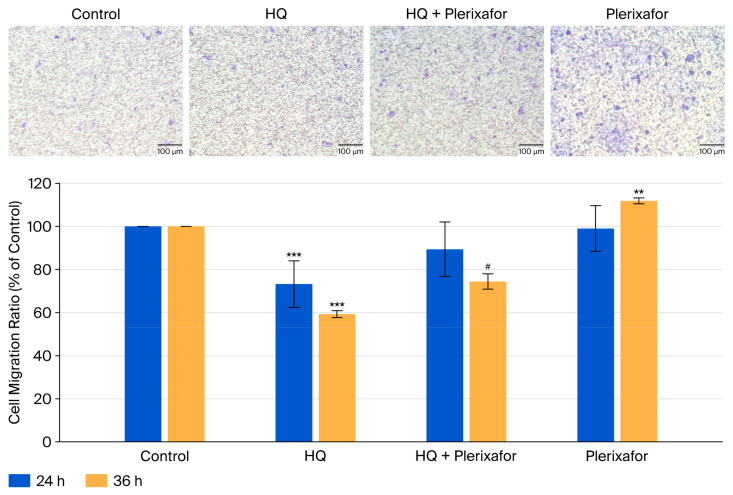
Plerixafor promotes PIG1 cell migration. Scale bar: 100 µm. The results are presented as the mean ± SD from *n* ≥ 5 independent experiments. Statistical significance was assessed by a one-way ANOVA followed by Tukey’s post hoc test. *p*-values are indicated as follows: ** *p* < 0.01, and *** *p* < 0.005 compared to the control; # *p* < 0.05 compared to the HQ group.

**Figure 3:**
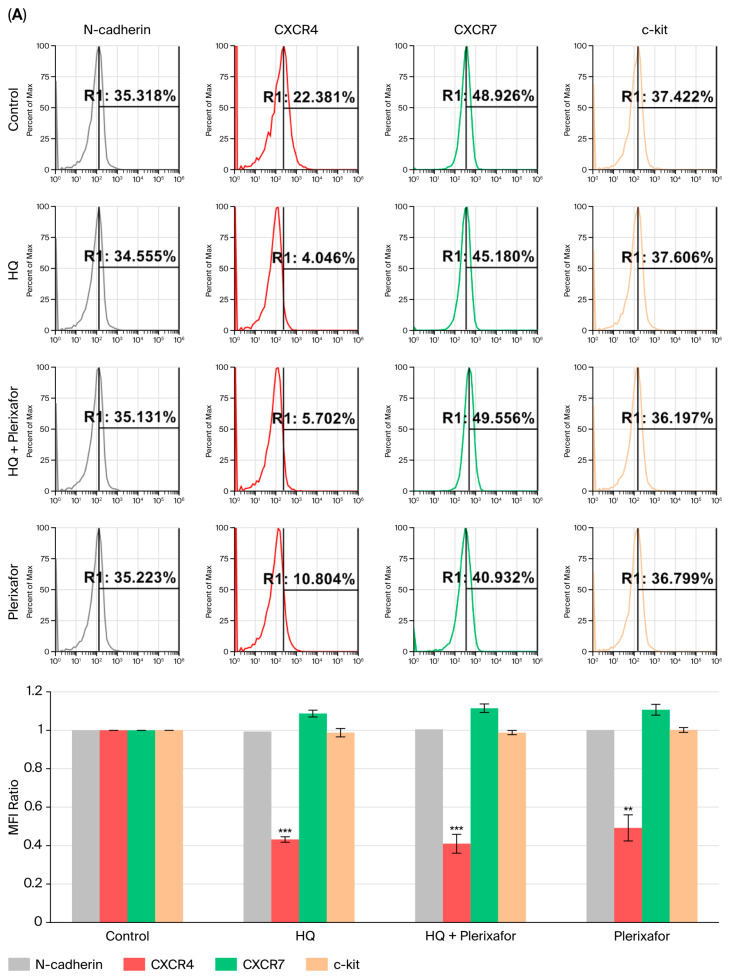

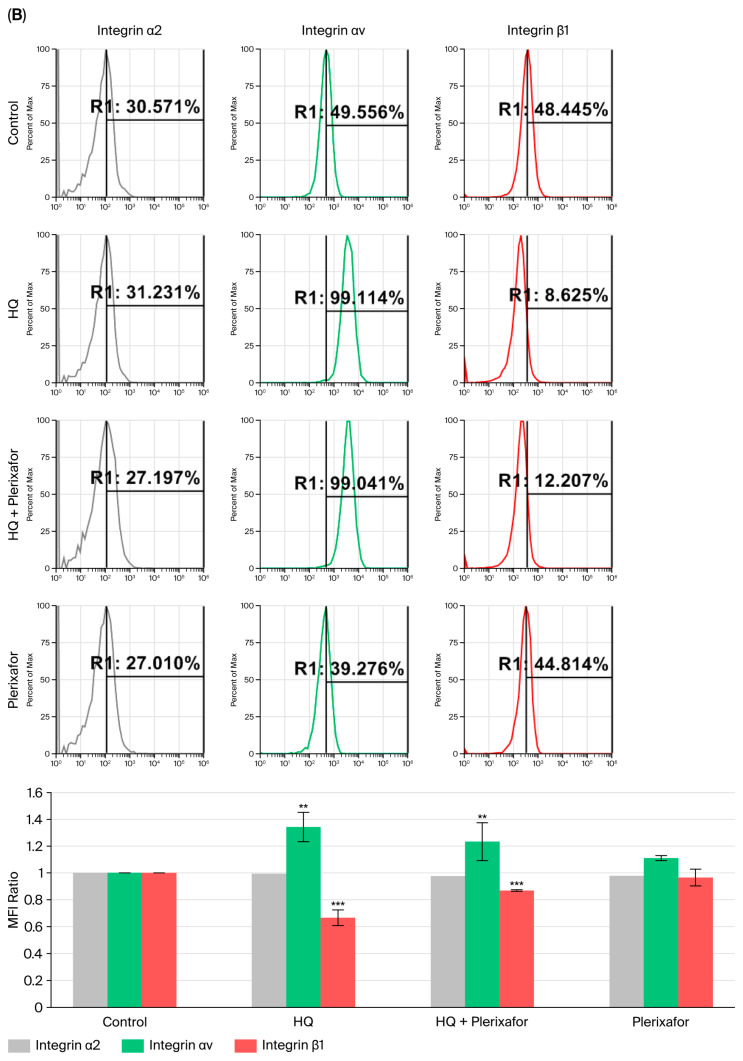
Plerixafor modulates integrin expression and CXCR receptor levels in PIG1 cells. (**A**) Representative histograms showing fluorescence intensity distributions for CXCR4, CXCR7, c-KIT, and N-cadherin; gray-lined histograms represent vehicle controls. (**B**) Integrin subunit expression (αv, α2, and β1). The right panels show quantification as fold change in mean fluorescence intensity (MFI) relative to controls. Data represent mean ± SD from *n* ≥ 3 independent experiments. Statistical significance was assessed by a one-way ANOVA followed by Tukey’s post hoc test. ** *p* < 0.01, and *** *p* < 0.001 vs. control; vs. HQ group.

Plerixafor enhanced the migration ability in PIG1 cells (Figure 2). Surface expressions of CXCR4, CXCR7, c-KIT, N-cadherin, and integrin subunits were assessed using flow cytometry. In control PIG1 cells, CXCR4-positive cells constituted approximately 23% of the population, which decreased significantly to 10% (*p* < 0.05) following Plerixafor treatment, consistent with receptor internalization [7]. In contrast, the rate of CXCR7-positive cells remained unchanged across treatment groups, indicating that Plerixafor selectively modulates CXCR4 (Figure 3A). For melanocyte lineage markers, c-KIT and N-cadherin expression remained stable, with no significant change following HQ or Plerixafor treatment.

Integrin profiling revealed that HQ treatment reduced the percentage of integrin β1-positive cells from 48% to 8% (*p* < 0.05). Conversely, integrin αv-positive cells increased from 49% to 99% following HQ treatment (*p* < 0.05), suggesting a shift in integrin heterodimer composition. Plerixafor did not significantly alter integrin β1 or αv expression compared with HQ-treated cells, indicating that Plerixafor-induced migration occurs independently of integrin modulation (Figure 3B). These results suggest that HQ shifts the balance of integrins β1/αv, which can influence cell motility; however, the actual outcomes depend on how the integrin is reconfigured and the specific composition of the extracellular matrix [26].

### 3.3. Plerixafor Enhances β-Catenin Activation

**Figure 4:**
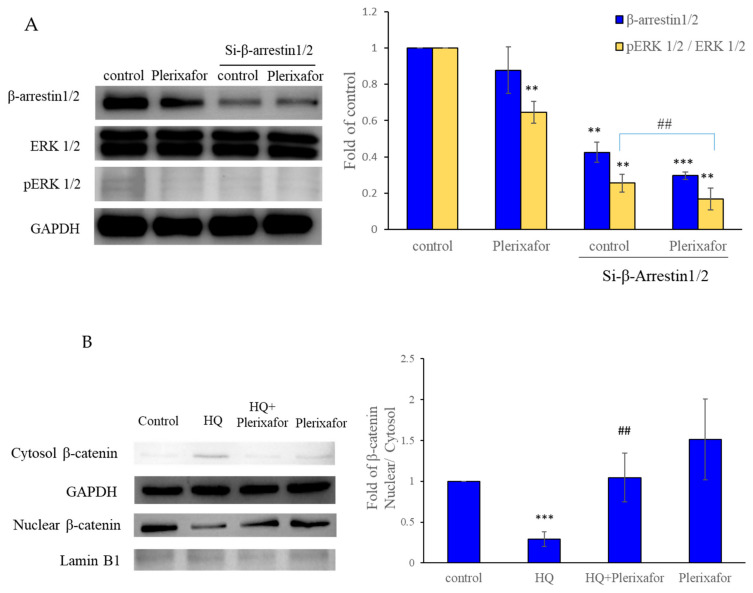

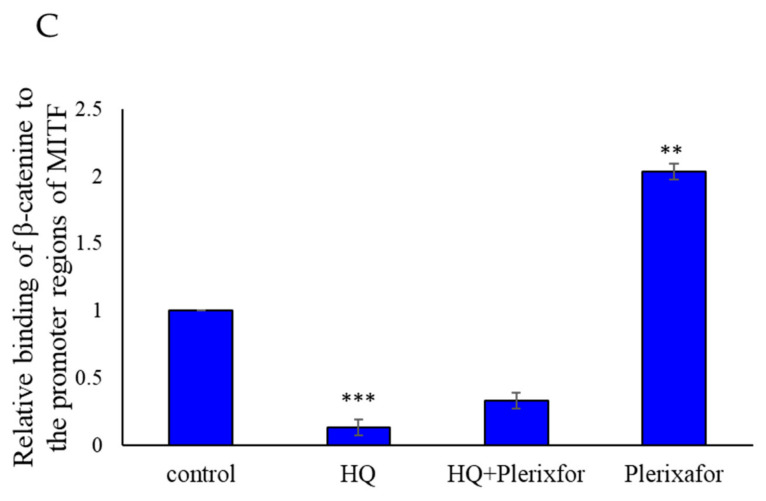
Plerixafor activates β-arrestin-dependent ERK and β-catenin–MITF signaling in PIG1 melanocytes. (**A**) Representative Western blots showing phosphorylated ERK1/2 (pERK) and total ERK1/2 (ERK) in PIG1 cells transfected with control siRNA or β arrestin1/2 siRNA and treated with Plerixafor or vehicle. GAPDH is shown as the loading control. The right panel shows densitometric quantification of the pERK/ERK ratio, normalized to the control group. (**B**) Western blot analysis of β-catenin in cytoplasmic and nuclear fractions from PIG1 cells treated with Plerixafor or vehicle. GAPDH and lamin B1 are shown as cytoplasmic and nuclear loading controls, respectively. (**C**) ChIP qPCR analysis showing β-catenin binding to MITF promoter/enhancer regions containing TCF/LEF motifs following Plerixafor treatment. Densitometric data (pERK/ERK ratios, β-catenin nuclear/cytoplasmic levels, and ChIP-qPCR enrichment) are presented as mean ± SD from at least *n* ≥ 5independent experiments. Comparisons between two groups (e.g., si-control vs. si-β-arrestin) were performed using Student’s *t*-test, and multiple-group comparisons were analyzed by a one-way ANOVA with Tukey’s post hoc test.** *p* < 0.01 and *** *p* < 0.001 vs. the control group. ## *p* < 0.01 vs. the HQ-treated group.

We further investigated the mechanism in PIG1 cells responding to Plerixafor, which reduces CXCR4-driven retention in combination with integrin modulation. Plerixafor exhibits biased antagonism at CXCR4 by selectively blocking G-protein-mediated signaling while still promoting β-arrestin recruitment to the receptor [27]. We use siRNA to knock down β-arrestin levels to evaluate pathway specificity in melanocytes in Plerixafor signaling. Transfection with si-β-arrestin markedly reduced β-arrestin protein levels, as confirmed by Western blotting (Figure 4A). β-Arrestin knockdown markedly reduces basal and Plerixafor-mediated ERK phosphorylation, indicating that when G-protein signaling is blocked by Plerixafor, any residual ERK activity is β-arrestin-dependent. Nevertheless, the loss of Plerixafor responsiveness in β-arrestin-deficient cells indicates a critical role of β-arrestin in this pathway.

Among key transcription factors binding the MITF promoter, a notable β-catenin translocation was detected in subcellular fractionation, compared with vehicle controls (Figure 4B). To determine the direct association with MITF regulatory sequences, we performed ChIP using anti-β-catenin antibodies, followed by qPCR with primers targeting the MITF promoter/enhancer regions that contain putative TCF/LEF binding motifs. ChIP-qPCR revealed a significant enrichment of β-catenin at these MITF regulatory loci in stimulated cells versus HQ (5- to 8-fold, *p* < 0.05), indicating that nuclear β-catenin directly binds to MITF-associated chromatin regions. These data showed that β-catenin translocation to the nucleus enables direct transcriptional regulation of MITF by β-catenin–TCF/LEF complexes, linking signaling to MITF-mediated transcriptional programs (Figure 4C).

### 3.4. Plerixafor Improved Re-Pigmentation in a Mouse Model Treated with HQ

**Figure 5:**
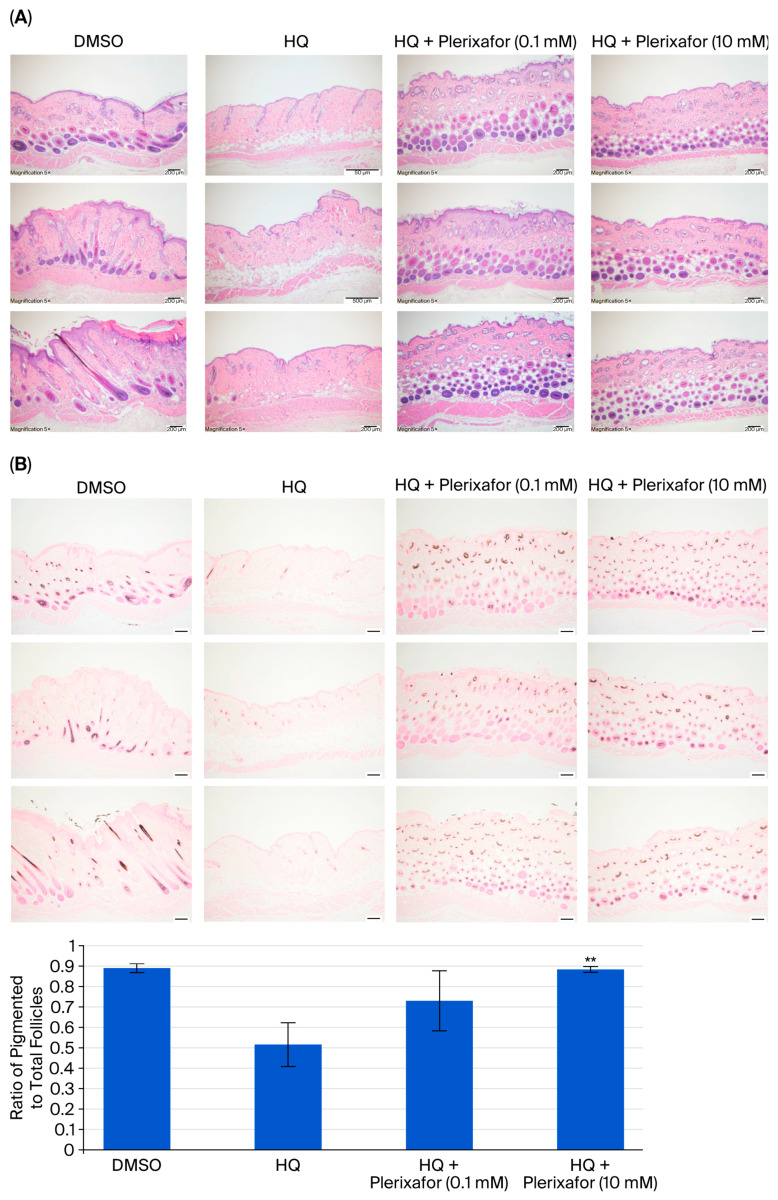

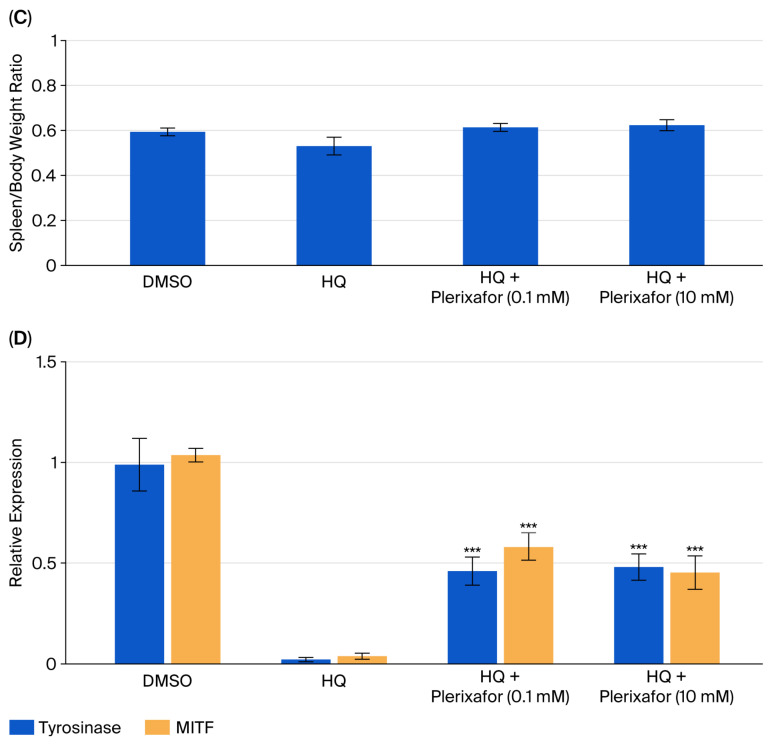
Plerixafor induces re-pigmentation in HQ-treated C57BL/6 mice. (**A**) Representative H&E-stained photographs. (**B**) Fontana–Masson-stained skin sections and quantified melanin content. Scale bar: 100 µm. (**C**) Spleen weight and body weight ratios. (**D**) The expression of the indicated genes in mouse skin tissue was measured by qPCR. Quantitative data for hair follicle counts, melanin content, spleen-to-body-weight ratios, and skin gene expression are presented as mean ± SD, with *n* = 5 mice per group. Group differences were analyzed by a one-way ANOVA followed by Tukey’s post hoc test. ** *p* < 0.01, and *** *p* < 0.005 compared to the HQ group.

Using HQ caused visible depigmentation on the backs of C57BL/6 mice. Topically applied Plerixafor improved re-pigmentation and increased the expression of MITF and tyrosinase in skin tissue. A significant difference in the number of hair follicles was observed between the experimental groups. Fontana–Masson staining was used to visualize and measure melanin, revealing that HQ eliminated hair follicles and their melanin content by approximately 50% compared with controls. In contrast, topical Plerixafor treatment restored the eliminated hair follicles, resulting in a 1.5-fold increase in their number and melanin content (*p* < 0.05), consistent with restoration of pigment production. Figure 5 provides the principal functional readout of pigment restoration in vivo, integrating follicular melanocyte activation and tissue-level melanin deposition in the HQ-treated mouse model. These structural changes did not differ following changes in concentration; it appears that the 0.1 mM topical dose is sufficient to affect melanogenesis and enhance hair follicle regeneration. Skin tissue analysis and HE staining revealed no local skin irritation or histologic inflammation. Biochemical tests revealed that serum aspartate aminotransferase (AST) and creatinine levels remained within normal ranges, confirming that the animal study did not cause liver or kidney damage and did not induce toxicity. The spleen-to-body-weight ratio showed no significant difference compared to the control group. This in vivo mouse model therefore, provides the primary tissue-level evidence that Plerixafor restores melanin deposition and re-pigmentation.

**Figure 6:**
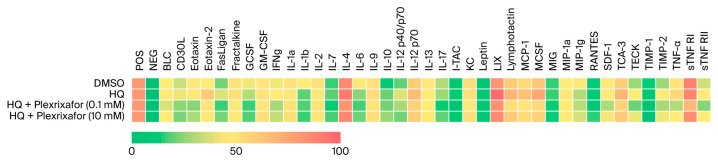
Effect of Plerixafor on proinflammatory cytokine protein levels in the serum of mice. Serum cytokine levels were profiled using an antibody array in *n* = 5 mice per group, and normalized signal intensities are displayed as heatmaps, with dark green indicating the lowest expression levels across the array and red the highest.

Cytokine antibody array analysis demonstrated that neither HQ alone nor Plerixafor, given individually or as an HQ + Plerixafor combination, altered the inflammatory cytokine profile compared with the control. Across all 40 proinflammatory cytokines measured, signal intensities in the Plerixafor group remained within the same range as those in the control groups, without consistent upward or downward trends, indicating that Plerixafor treatment, alone or combined with HQ, did not measurably modulate this group of inflammation-related mediators under the tested conditions (Figure 6).

## 4. Discussion

In this study, we identify Plerixafor as a CXCR4-biased ligand that promotes melanogenesis through β-arrestin-dependent signaling and β-catenin–MITF transcriptional regulation in melanocytes. Plerixafor is clinically used as a CXCR4 antagonist for hematopoietic stem cell mobilization, but its biased actions and downstream signaling consequences in melanocytes have not been defined. Elucidating how Plerixafor reprograms CXCR4 signaling at the biochemical and molecular levels, and how this translates into changes in melanocyte behavior and skin pigmentation, is therefore of interest for both GPCR biased-signaling pharmacology and drug repurposing. Our results show that Plerixafor, a CXCR4 antagonist with biased signaling properties, increases MITF and tyrosinase expression in human melanocytes, inducing a strong pro-melanogenic effect and directly supporting its role in promoting melanogenic gene programs. Importantly, Plerixafor counteracted the HQ-induced suppression of melanogenesis, restoring MITF and tyrosinase expression and providing a biological rescue against depigmentation agents. Available clinical reports do not consistently link systemic Plerixafor to recognizable pigmentary effects; however, rare, delayed, or tissue-specific pigment changes cannot be ruled out, especially in view of its short-term use for hematopoietic stem cell mobilization.

Beyond pigmentation, our data raise the possibility that Plerixafor may influence hair follicle dynamics. Melanocyte stem cells serve as the source of melanocyte replenishment in adult skin. They are found in specific skin niches, such as the hair follicle bulge region, the epidermal basement membrane, and sweat glands [28]. Homeostasis of melanocytes depends on the closely coordinated signaling of melanocyte stem cells, paracrine factor communication, transcriptional networks, and immune/stress responses to regulate melanocyte survival, quantity, and melanin production, thereby maintaining healthy skin pigmentation [29]. Further research is necessary to understand how recruiting these progenitor cells in specific situations can provide pigment cell sources for re-pigmenting depigmented areas. Yamada, T. et al. reported that CXCL12 attracts melanocyte stem cells to the proper position, facilitating the maintenance of their undifferentiated state [30]. The same team reported that differentiation of melanocytes occurs through the activation of β-catenin and migration to the epidermis to complete re-pigmentation in mice [31]. Our study showed that the CXCL12-CXCR4 axis antagonist Plerixafor activates a β-arrestin-dependent signaling pathway associated with β-catenin nuclear translocation, interaction with T-cell-specific factor/lymphoid enhancer-binding factor 1 (TCF/LEF-1), and binding to the TCF/LEF-1-responsive region of the MITF promoter, suggesting that Plerixafor diminishes the CXCL12-mediated retention signal and promotes melanogenic differentiation-associated signaling, but the present study is not sufficient to define true follicle regeneration or specific hair-cycle transitions. Future work will therefore require time-course analyses with established markers of hair-cycle phase (such as Ki67 and canonical anagen/telogen markers), quantitative hair-cycle staging across multiple time points, and, ideally, lineage-tracing approaches to distinguish newly generated follicles from activation of pre-existing follicular pigment cells. These experiments will be essential to determine whether Plerixafor genuinely promotes hair follicle regeneration or primarily enhances melanocyte activation and melanin production within existing follicles.

β-arrestin is essential for Plerixafor signaling in melanocytes, as evidenced by the complete loss of Plerixafor effects when β-arrestin was silenced. In this study, a dual siRNA approach targeting β-arrestin1 and β-arrestin2 was used to obtain a more robust knockdown than either single siRNA alone. Future work will incorporate complementary approaches, such as alternative siRNA sequences or rescue experiments, to further validate knockdown specificity. Plerixafor affects the CXCL12-CXCR4–ERK pathway by fully blocking G-protein activation at CXCR4, while simultaneously influencing a β-arrestin-biased route that supports alternative downstream β-catenin/MITF without restoring canonical ERK signaling. Future studies dissecting alternative β-arrestin-scaffolded effectors (e.g., PI3K, Src, and Rho GTPases) [32] may clarify which downstream nodes drive melanogenesis versus migration. The nuclear translocation of β-catenin, observed through subcellular fractionation and ChIP-qPCR analysis, reveals a key downstream mechanism by which Plerixafor boosts MITF transcriptional activity. β-Catenin directly binds to the TCF/LEF motif [CTTTGAT] located near the transcriptional start site on the MITF promoter/enhancer, thereby linking CXCR4 antagonism to canonical Wnt signaling pathways that promote melanogenic gene expression.

Beyond melanogenesis, Plerixafor influenced melanocyte migration, suggesting that CXCR4 signaling also regulates pigment cell positioning. A previously reported study revealed that CXCL12 binding to CXCR4 receptors induces the activation of integrins [33]. In essence, Plerixafor’s effect on cell migration was supported by increased motility in PIG1 cells and independent of changes in the expression of melanocyte surface markers c-KIT and N-cadherin. Notably, Plerixafor reduced CXCR4 surface expression, consistent with prior studies showing receptor internalization driven by β-arrestin recruitment under biased antagonism. CXCR7 levels remained stable, underscoring a selective modulation of CXCR4 by Plerixafor. Additionally, HQ treatment reduced the surface levels of integrin β1. It concomitantly increased integrin αv expression, consistent with a shift in available heterodimer pairs toward αv-containing receptors when β1 is limiting, as described for epithelial and carcinoma cells [26]. This pattern is compatible with altered adhesion to fibronectin-rich or vitronectin-rich matrices and, in principle, could influence melanocyte anchorage and migration [34]. Nevertheless, integrin regulation appears to play a permissive rather than primary role in the pro-migratory effects of Plerixafor. Although both HQ and Plerixafor alone induced a comparable reduction in CXCR4 at the cell surface, their downstream consequences for melanocyte behavior appear to be mechanistically distinct. In our system, HQ mainly disrupts the αv/β1 integrin balance, altering cell–matrix interactions and creating a primed state that is more responsive to chemokine cues, but it does not strongly activate CXCR4–β-arrestin or ERK/β-catenin–MITF signaling. By contrast, Plerixafor functions as a β-arrestin-biased CXCR4 ligand, driving robust β-arrestin-dependent ERK activation and β-catenin–MITF signaling to enhance melanocyte motility even under HQ-induced integrin imbalance. Rather than reshaping integrin expression, Plerixafor appears to exploit this HQ-sensitized background to promote CXCR4–β-arrestin-dependent migration, a crosstalk that will require future studies using integrin-blocking antibodies or defined ECM substrates to delineate in detail.

The findings should be interpreted in light of the limitations of the experimental models used. In our system, HQ decreased the expression of melanogenic genes, reduced CXCR4 surface levels, and altered cytoskeletal organization, thereby changing melanocyte motility, which is consistent with previous reports [35,36]. While Plerixafor-induced CXCR4 downregulation can be attributed to β-arrestin-mediated receptor internalization, the mechanism underlying HQ-induced CXCR4 reduction requires further investigation, although it may involve altered receptor stability under oxidative stress. Because PIG1 cells contain little to no detectable melanin, they were used in this study primarily as a mechanistic human melanocyte model for transcriptional and signaling analyses, and a 2% hydroquinone solution was applied to mice to reliably induce stable hypopigmentation of the hair and skin for tissue-level evaluations of pigment restoration. Given that Plerixafor treatment generated round, melanin-containing hair follicles, Plerixafor may be a potential candidate for promoting hair follicle regeneration, maturation of melanogenic progenitor cells, and melanin production in both hair follicles and the epidermis. In addition, this in vivo study demonstrated consistent pigmentary efficacy at two distinct topical doses of Plerixafor (0.1 mM and 10 mM), both of which improved re-pigmentation and increased melanogenic readouts under the present experimental conditions. Although these data support reproducible biological activity across a 100-fold dose range, a full dose–response analysis will still be required in future studies to more precisely define the minimum effective dose and therapeutic window. Additionally, the WNT/β-catenin signaling pathway is one of the earliest and most critical pathways for hair follicle induction [37]. In this study, topical Plerixafor at the tested doses did not substantially alter serum AST or creatinine levels, spleen-to-body-weight ratios, or profiles of measured inflammatory cytokines under the experimental conditions. Histological examination of treated skin also did not reveal local inflammatory infiltrates or tissue damage, indicating that Plerixafor’s pigmentary and hair follicle effects occur without clear histopathological evidence of dermal toxicity in this model. The functional role of Plerixafor related to hair follicles remains an area in need of in-depth exploration and explanation, and more comprehensive toxicology studies will be required to rigorously define the safety profile of topical Plerixafor for the future.

## 5. Conclusions

In summary, this study demonstrates that Plerixafor enhances melanogenesis-related responses through a β-arrestin-dependent CXCR4 signaling mechanism associated with β-catenin-mediated transcriptional regulation of MITF. Activation of this significant pathway increased melanogenesis-associated gene expression, promoted melanocyte migration, and partially neutralized depigmentation in our experimental models. These results pinpoint a previously unrecognized role for biased CXCR4 signaling in melanocyte function and suggest that Plerixafor may represent a potential candidate for further investigation in re-pigmentation strategies. However, the therapeutic implications of these findings remain preclinical and hypothesis-generating, as the current study is based on mechanistic analyses in PIG1 melanocytes and a preclinical mouse depigmentation model. Direct assessment of melanin production in human melanocytes and validation using human-relevant skin models will be required to further establish the translational potential of Plerixafor. In addition, future studies should investigate whether Plerixafor can produce clinically relevant pigmentary effects, define its safety profile, and clarify the contribution of CXCR4 signaling and integrin-mediated pathways in melanocyte migration.

## Data Availability

The original contributions presented in this study are included in the article/Supplementary Material. Further inquiries can be directed to the corresponding author(s).

## Supplementary Materials

The following supporting information can be downloaded at: https://www.mdpi.com/article/10.3390/cimb48070730/s1.

## Abbreviations

The following abbreviations are used in this manuscript:

HQ	Hydroquinone
Tyr	Tyrosinase
MITF	Microphthalmia-associated transcription factor
PMEL	Premelanosome protein
GAPDH	Glyceraldehyde 3-phosphate dehydrogenase
