# Supplementary material for: Plerixafor Engages β-Arrestin-Dependent CXCR4 Signaling to Promote Melanogenesis via β-Catenin-MITF Activation
Source: Curr Issues Mol Biol. 2026 Jul 17;48(7):730. doi: 10.3390/cimb48070730 (PMC13407746; doi:10.3390/cimb48070730)
Supplement: Supplementary file 1 [file cimb-48-00730-s001.zip › cimb-4376482-supplementary.pdf]

Table S1. Primer sequences used in this study.

Species	Primers	Nucleotide Sequence 5′ to 3′
Mouse	TYR-F	ACAAATACATACCTTGAACCGCT
	TYR-R	CCCTTTATCACAATACAATTCCCTC
	MIFT-F	CATCATCAGCCTGGAATCAAGT
	MIFT-R	GAGGTCGATCAAGTTTCCAGAGA
	GAPDH-F	TGAGGCCGGTGCTGAGTATGTC
	GAPDH-R	CCACAGTCTTCTGGGTGGCAGTG
Human	TYR-F	CAGTTCCTGCAGACCTTGTGAG
	TYR-R	AGGAGACACAGGCTCTAGGGA
	MIFT-F	TTATTCCATCCACGGGTCTCTGC
	MIFT-R	CTGCATGATGCTGAAGGAGGTC
	GAPDH-F	TGAGGCCGGTGCTGAGTATGTC
	GAPDH-R	CCACAGTCTTCTGGGTGGCAGTG
	18S rRNA-F	GGCCCTGTAATTGGAATGAGTC
	18S rRNA-R	CCAAGATCCAACTACGAGCTT

Table S2. ChIP primers numbered according to the reference sequence NG_050802.1.

Human MITF	Nucleotide Sequence 5′ to 3′	Location	Amplicon Size (104 bp)
Forward primer	GCCTTGATCTGACAGTGAGTTTG	2067–2089	GCCTTGATCTGACAGTGAGTTTGA**[CTTTGAT]**AGCTCGTCACTTAAAAAGGTTCTTTTATATTTATGAAAAAAAGCATGACGTCAAGCCAGGGGGAAAAATTGA
Reverse primer	TCAATTTTTCCCCCTGGCTTGA	2169–2148	
